# Retraction Note: Diacerein ameliorates amiodarone-induced pulmonary fibrosis via targeting the TGFβ1/α-SMA/Smad3 pathway

**DOI:** 10.1007/s00210-026-05324-7

**Published:** 2026-04-11

**Authors:** Hadir Farouk, Passant E. Moustafa, Marwa S. Khattab, Salma A. El-Marasy

**Affiliations:** 1 Department of Pharmacology, Medical Research and Clinical Studies Institute, National Research Centre, Giza, Egypt; 2 Department of Pathology, Faculty of Veterinary Medicine, Cairo University, Giza, Egypt

**Retraction Note: Naunyn-Schmiedeberg's Archives of Pharmacology (2024) 398:4111-4122**

10.1007/s00210-024-03450-8

The Editor-in-Chief has retracted this article. After publication, concerns were raised regarding the size of the tidal volumes used, which were not perceived to be reasonable in the animal model used. The Authors were unable to provide a sufficient response to concerns. The Editor-in-Chief has lost confidence in the data and conclusions of this article.

Authors Marwa S Khatta, Salma A. El-Marasy, Hadir Farouk and Passant Elwy Moustafa agree with this retraction.

